# Chemotherapy Plus Best Supportive Care versus Best Supportive Care in Patients with Non-Small Cell Lung Cancer: A Meta-Analysis of Randomized Controlled Trials

**DOI:** 10.1371/journal.pone.0058466

**Published:** 2013-03-13

**Authors:** Chenxi Zhong, Hongcheng Liu, Liyan Jiang, Wei Zhang, Feng Yao

**Affiliations:** 1 Department of Thoracic Surgery, Shanghai Chest Hospital affiliated to Shanghai Jiao Tong University, Shanghai, China; 2 Department of Thoracic Surgery, Shanghai Pulmonary Hospital, School of Medicine, Tongji University, Shanghai, China; 3 Department of Pulmonary Medicine, Shanghai Chest Hospital affiliated to Shanghai Jiao Tong University, Shanghai, China; Memorial University of Newfoundland, Canada

## Abstract

**Background:**

The use of chemotherapy has been proposed to increase the effectiveness of best supportive care (BSC) in patients with non-small cell lung cancer (NSCLC). Previous trials reported inconsistent findings regarding the efficacy and safety of chemotherapy on overall survival (OS) and treatment-related mortality. We performed a systematic review and meta-analysis to evaluate the effects of chemotherapy plus BSC versus BSC alone on survival of patients with NSCLC.

**Methodology and Principal Findings:**

We systematically searched PubMed, EmBase, and the Cochrane Central Register of Controlled Trials for relevant literature. All eligible studies included patients with NSCLC who had received chemotherapy and BSC or BSC alone. All eligible studies measured at least 1 of the following outcomes: OS or treatment-related mortality. Overall, patients that received chemotherapy plus BSC had significant longer OS than those that received BSC alone (HR, 0.76; 95%CI, 0.69–0.84; P<0.001). Additionally, chemotherapy plus BSC as compared to BSC alone resulted in a 28% RR reduction (95%CI: 12–40; P = 0.001) in 6-month mortality, 11% RR reduction (95%CI: 8–15; P<0.001) in 12-month mortality, and 5% RR reduction (95%CI: 1–8; P = 0.02) in 2-year mortality. Toxicity was greater in patients that received chemotherapy plus BSC.

**Conclusion/Significance:**

Chemotherapy plus BSC increased the OS and reduced the 6-month, 12-month, and 2-year mortality of NSCLC patients.

## Introduction

Lung cancer is the leading cause of cancer death worldwide for both men and women. Around one and a half million new cases are diagnosed each year. NSCLC accounts for approximately 80–85% of all lung cancer cases and is the most common cause of cancer death in industrialized countries [1]–[2]. Surgery is generally regarded as the best treatment option; however, only approximately 30% of lung cancers are suitable for potentially curative resection. A further 20% of patients with locally advanced disease undergo radical thoracic radiotherapy or concurrent chemo-radiotherapy. The remaining 50% of patients with metastatic disease received BSC, and it has been suggested that addition of chemotherapy to BSC may offer further benefits [3].

Historically, standard chemotherapy has provided modest improvements to OS, and chemotherapy-treated patients scored better on the quality of life functioning scale than patients receiving BSC alone [4]–[6]. Previous trials [7] reported fewer lung cancer-related symptoms but worse toxicity-related symptoms, and the median survival time and 1-year survival rate with chemotherapy were prolonged to 8–10 months and 30–35%, respectively. Another study demonstrated that chemotherapy has improved progression-free survival, but that the effectiveness was limited or negated by its toxicity [8].

Here, we determined the effectiveness of chemotherapy in patients with NSCLC as compared to BSC in terms of OS, treatment-related mortality, and drug-related adverse events. We carried out a systematic review and meta-analysis of pooled data from randomized controlled trials to evaluate the effects of chemotherapy plus BSC on survival of patients with NSCLC.

## Methods

### Data sources, search strategy, and selection criteria

We adapted the Cochrane Central Register of Controlled Trials, Medline, and EmBase, with relevant text words and medical subject headings that included all spellings of chemotherapy agents, “chemotherapy,” “non-small cell lung cancer,” “NSCLC,” “randomized controlled trials,” “human,” and “English.” Reference lists from identified trials and review articles were manually scanned to identify any other relevant studies. Furthermore, we also searched http://www.ClinicalTrials.gov for information on registered randomized controlled trials to identify trials that were registered as completed, but whose results had not yet been published. This review was conducted and reported according to the Preferred Reporting Items for Systematic Reviews and Meta-Analysis (PRISMA) Statement issued in 2009 (Table S1) [9].

References identified by the search strategy were screened independently by 2 authors (CZ and HL) to evaluate their eligibility for inclusion in our meta-analysis. Any disagreement between these 2 reviewers was settled by the third reviewer (FY) until a consensus was reached. All completed randomized controlled trials that evaluated the effects of chemotherapy on survival of patients with NSCLC were eligible for inclusion. The analyzed outcomes were OS, treatment-related mortality, and any possible drug-related adverse events.

### Data collection and quality assessment

For each of the included trials, details of the treatment given and outcomes were recorded independently by 2 authors (JL and WZ) and any disparities were resolved by a group discussion. Data were extracted from the included trials in terms of patient characteristics, intervention, and methodological characteristics of the included trials. The primary reported outcomes were summarized in tables. One author (JL) entered the data into the computer and another author (FY) checked it. The outcomes investigated included OS, treatment-related mortality, and any possible drug-related adverse events. The quality of the study was assessed using the Jadad score [10] (CZ) on randomization, concealment of the treatment allocation, blinding, completeness of follow-up, and the use of intention-to-treat analysis.

### Statistical analysis

The effect of treatment was estimated using hazard ratios (HRs) and risk ratios (RRs) with their corresponding confidence intervals (CIs). For time-to-event data, the log HRs and their variances were estimated using the methods proposed by Parmar [11] when CIs of HRs were reported. The summary HRs and their 95% CIs were estimated using a general variance-based method. RRs were computed for dichotomous variables, estimates of the treatment effects were obtained from the number of events reported in each arm and combined using the methods reported by Mantel and Haenszel [12]. The drug-related adverse events were analyzed as WHO grades 3 or more. We explored potential heterogeneity in estimates of treatment effect with univariate meta-regression for baseline characteristic of patients with NSCLC. After this, we performed subgroup analysis to explore potential effect on OS, the 12-month and 2-year mortality based on the number of patients, mean age, proportion of male patients, type of chemotherapy, and the Jadad score. All estimates of effects were derived using a random-effects model [13]–[14]. Heterogeneity of the treatment effects between studies was investigated visually by a scatter plot and statistically by the heterogeneity I^2^ statistic [15]. Egger test was used to check for potential publication bias [16]. All the reported p values are 2-sided, and p values of <0.05 were regarded as statistically significant for all included studies. All analyses were calculated using the software STATA (version 10.0).

## Results

**Figure 1:**
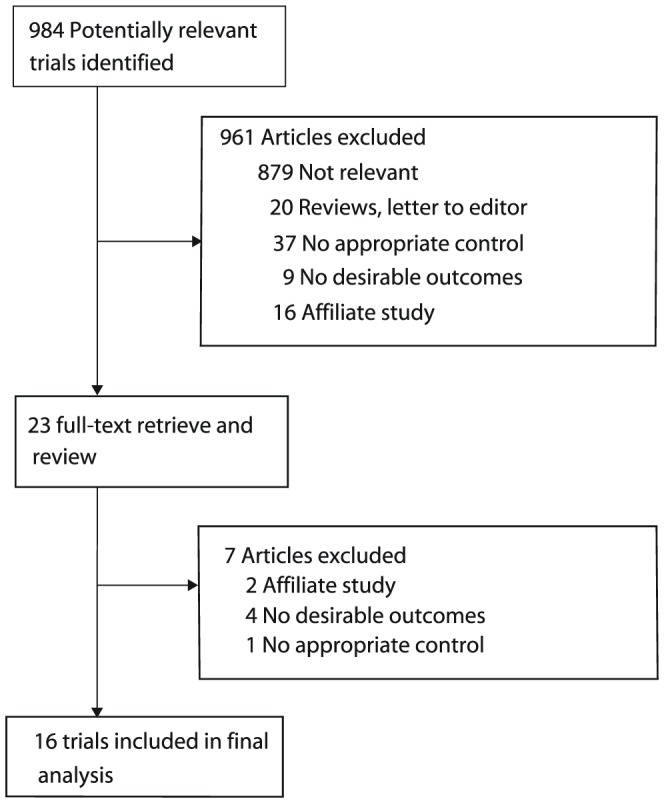
Identification process for eligible studies.

We identified 984 potential articles from our initial electronic search, of which 961 were excluded during an initial review of titles and abstracts. We retrieved full texts of the remaining 23 studies and 16 randomized controlled trials [8], [17]–[32] met the inclusion criteria (Figure 1 and Figure S1). Nine out of the included trials [19], [22], [26]–[32] were evaluating platinum-based chemotherapy plus BSC compared with BSC alone, 2 [17]–[18] were assessing gemcitabine or vinorelbine therapy plus BSC compared with BSC alone, 3 [20], [21], [25] were evaluating taxel therapy compared with BSC alone, and the remaining 2 [23], [24] assessed the effects of pemetrexed therapy compared with BSC alone. Most of other studies identified by our search did not examine the effects of chemotherapy, or did not compare different treatments. Furthermore, some trials were not original investigations, or were duplicates of reports that had already been published [33], [34]. Our final analysis of 16 trials included a total of 4,135 patients with NSCLC. Table 1 summarizes characteristics of patients included in the trials. The trials had a sample size that ranged from 48–725 patients, with a mean of 258 patients. Data for OS were available in 13 trials [17]–[19], [21], [22], [24], [26]–[32], for 6-month mortality in 4 trials [18], [23], [27], [29], for 12-month mortality in 11 trials [17], [18], [20]–[22], [25]–[27], [29], [31], [32], and for 2-year mortality in 7 trials [17], [22], [25]–[27], [31], [32]. Reporting of key indicators of trial quality was scarce, with earlier studies providing few details about the process of randomization, concealment of allocation, and the use of intention-to-treat analysis. The quality of the trials was also assessed by pre-defined criteria using Jadad score [10]. Overall, out of the 16 trials, 1 scored 5 [24], 4 scored 4 points [18], [20], [22], [23], 4 scored 3 points [17], [21], [26], [28], 6 scored 2 points [19], [25], [27], [29], [30], [32] and 1 trial scored 1 point [31] on the Jaded score.

**Table 1:** Design and characteristic of trials included in our meta-analysis.

Source	No. of patients	Sex (male, %)	Mean age, y	Stage of disease	Intervention	Jadad score
H Anderson [17]	300	63.3	64.5	Locally advanced and metastatic NSCLC	Gemcitabine plus BSC; BSC	3
The ELCVIS Group [18]	154	87.0	74.0	IIIB or IV NSCLC	Vinorelbine; BSC	4
RL Woods [19]	188	81.9	61.0	Advanced NSCLC	Cisplatin and vindesine; BSC	2
By Frances A [8], [20]	204	67.2	61.0	IIIA, IIIB or IV NSCLC	Docetaxel; BSC	4
M Ranson [21]	157	75.0	64.0	IIIB or IV NSCLC	Paclitaxel Plus BSC; BSC	3
SG Spiro [22]	725	65.5	74.0	Advanced NSCLC	cisplatin-based chemotherapy plus BSC; BSC	4
L Paz-Ares [23]	539	58.1	61.3	IIIB or IV NSCLC	Pemetrexed plus BSC; BSC	4
T Ciuleanu [24]	663	73.0	60.5	IIIB or IV NSCLC	Pemetrexed plus BSC; placebo plus BSC	5
K Roszkowski [25]	207	81.6	59.3	metastatic or non- resectable localized NSCLC	Docetaxel plus BSC; BSC	2
M Helsing [26]	150	59.0	64.0	Advanced NSCLC	Carboplatin, Etoposide plus BSC; BSC	3
G Cartel [27]	102	73.0	56.6	Stage IV NSCLC	Cisplatin, cyclophosphamide, mitomycin plus BSC; BSC	2
S Kaasa [28]	87	79.3	62.0	Inoperable, extensive NSCLC	Cisplatin, etoposide; symptomatic treatment	3
**B**R Cellerino [29]	123	96.7	60.5	Advanced NSCLC	Cyclophosphamide, epirubicin, cisplatin, methotrexate, etoposide, and lomustine; BSC	2
PA Ganz [30]	48	89.6	NG	advanced metastatic NSCLC	Cisplatin, vinblastine plus BSC; BSC	2
**B**E Rapp [31]	137	74.5	NG	Advanced NSCLC	vindesine and cisplatin/cyclophosphamide, doxorubicin, and cisplatin; BSC	1
MH Cullen [32]	351	72.4	63	Unresectable NSCLC	Mitomycin, ifosfamide, cisplatin plus palliative care; palliative care	2

**Figure 2:**
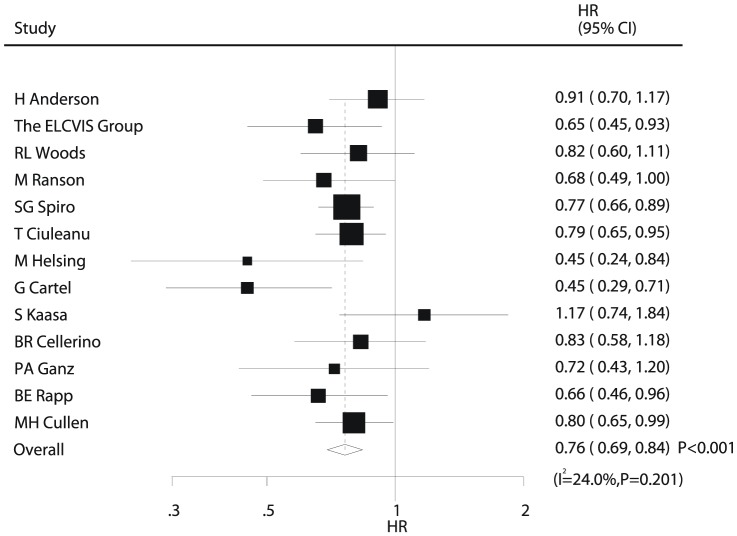
Comparison of overall survival between chemotherapy plus best supportive care and best supportive care alone.

Data for OS were available from 13 trials [17]–[19], [21], [22], [24], [26]–[32]. Data from trials by Frances A [8], L Paz-Ares [22], and K Roszkowski [24] were excluded from the analysis of OS in our study because the authors did not provide this information. Overall, we concluded that chemotherapy plus BSC yielded a clinically and statistically significant 24% improvement in OS compared with BSC alone (HR, 0.76; 95%CI, 0.69–0.84; P<0.001, Figure 2). Although there was some evidence of heterogeneity across the trials included (I^2^ = 24%, P = 0.201), a sensitivity analysis indicated that the results were not affected by sequential exclusion of a particular trial from the pooled analysis.

**Figure 3:**
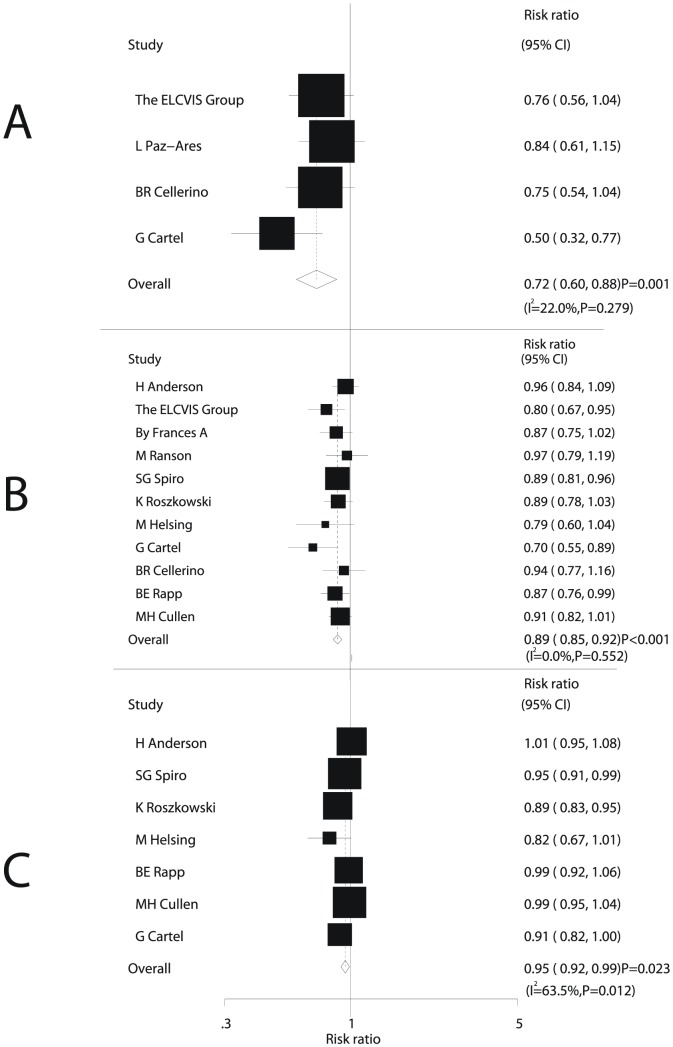
Comparison of 6-month mortality (A), 12-month mortality (B), and 2-year mortality (C) between chemotherapy plus best supportive care and best supportive care alone.

Data for the effect of chemotherapy on the 6-month mortality were available in 4 trials [18], [23], [27], [29], including 918 patients and 320 events of death. Overall, we noted that chemotherapy plus BSC resulted in a 28% reduction in the risk of 6-month mortality compared with BSC alone (RR, 0.72; 95%CI, 0.60–0.88; P = 0.001, Figure 3A). Additionally, we noted some evidence of heterogeneity in the magnitude of the effect across the included trials (I^2^ = 22%, P = 0.279); however, after sequential exclusion of trials from the pooled analysis, the results were not affected by exclusion of a specific trial.

The risk of 12-month mortality was reported in 11 trials [17], [18], [20]–[22], [25]–[27], [29], [31], [32], which included 2520 patients and recorded 1932 events of death. Overall, chemotherapy plus BSC reduced the risk of 12-month mortality by 11% without evidence of heterogeneity (RR, 0.89; 95%CI: 0.85–0.92, P<0.001, Figure 3B).

Seven trials [17], [22], [25]–[27], [31], [32], which provided data for the 2-year mortality, included 1883 patients and 1764 events of death. Overall, chemotherapy plus BSC reduced the risk of 2-year mortality by 5% (RR, 0.95; 95%CI, 0.92–0.99; P = 0.02, Figure 3C). However, because we noted a substantial heterogeneity in the RRs for the 2-year mortality from the individual trials (I^2^ = 63.5%, P = 0.012), we performed a subgroup analysis based on the number of patients, mean age, proportion of male, interventions, and Jadad score, to explore potential contributing factors.

We noted several adverse events reported by a few trials. Overall, we noted that treatment with chemotherapy plus BSC were associated with significant increase in the risks of neutropenia, leukopenia, anemia, infection, nausea/vomiting, alopecia, and ankle swelling (Table 2). No other significant differences were identified between the effects of chemotherapy plus BSC and BSC alone.

**Table 2:** Summary of the relative risks of grade 3 or worse toxicity assessed.

Outcomes	Included studies	RR and 95% CI	P value	Heterogeneity (%)	P value for heterogeneity
Neutropenia	7	31.01 (10.71–89.75)	<0.001	0	0.73
Leukopenia	6	11.49 (3.50–37.69)	<0.001	14	0.32
Thrombocytopenia	4	4.10 (0.91–18.51)	0.07	0	0.97
Anemia	6	3.85 (1.58–9.38)	0.003	12	0.34
Infection	7	2.10 (1.04–4.25)	0.04	10	0.36
Nausea and vomiting	9	3.82 (1.31–11.14)	0.01	47	0.06
Asthenia	6	1.97 (0.74–5.25)	0.18	81	<0.001
Rash	2	0.85 (0.49–1.46)	0.55	0	0.72
Pulmonary toxicity	4	0.64 (0.29–1.42)	0.27	58	0.07
Alopecia	3	15.84 (1.05–239.49)	0.05	80	0.007
Ankle swelling	2	2.64 (1.61–4.33)	<0.001	0	0.96
Constipation	2	7.38 (0.95–57.05)	0.06	0	0.78
Cardiac toxicity	3	1.14 (0.22–6.01)	0.88	0	0.95
Neuromotor	3	3.76 (0.23–61.10)	0.35	71	0.03
Diarrhea	3	4.15 (0.72–23.97)	0.11	0	0.81
Stomatitis/Mucositis	5	3.12 (0.79–12.32)	0.10	0	0.98
Neurosensory	4	1.33 (0.25–7.01)	0.74	35	0.20
Anorexia	2	3.97 (0.47–33.31)	0.20	0	0.41
Peripheral neuropathy	2	7.36 (0.92–59.00)	0.06	0	0.70

**Figure 4:**
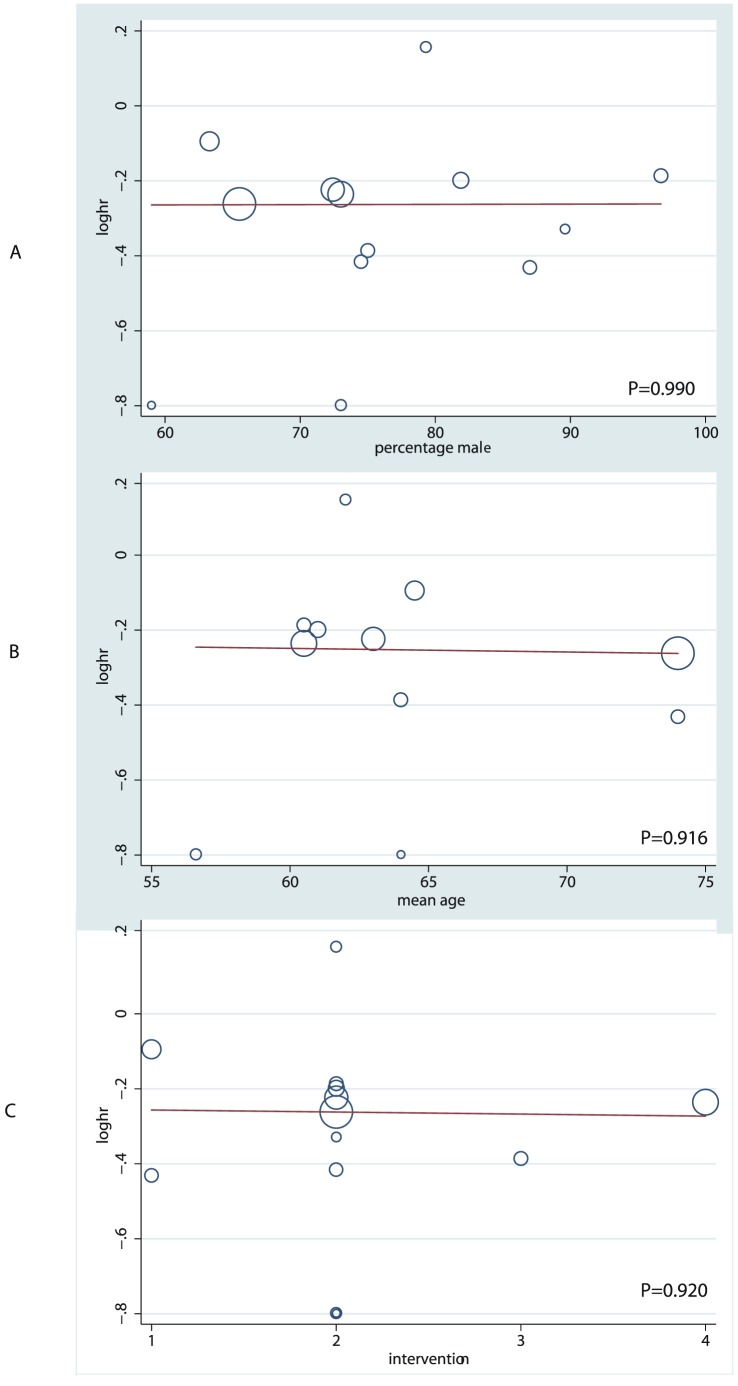
Meta-regression of (A) percentage male, (B) mean age, and (C) interventions for overall survival.

In an exploratory attempt to identify sources of the residual differences between trials, we performed meta-regression analysis of the mean age, proportion of men, and interventions for OS. However, these variables did not appear to be important contributing factors to the effect of chemotherapy plus BSC for OS (Figure 4).

Subgroup analyses were done for OS, 12-month mortality, and 2-year mortality. Overall, we noted that chemotherapy (except gemcitabine/vinorelbine treatment) was associated with clinically and statistically significant improvement in OS as compared to BSC (Table 3). Similarly, we also observed that chemotherapy, except gemcitabine/vinorelbine, had a clear effect on the 12-month mortality(Table 3). Finally, we noted that chemotherapy was associated with reduced risk of 2-year mortality, when in percentage male greater than 80% and with platinum-based chemotherapy and taxel, and in trials with the Jadad score of 4 or 5 points (Table 3). No other significant differences were identified between the effects of chemotherapy and BSC, when based on additional subset factors (Table 3).

**Table 3:** Subgroup analysis of overall survival, 12-month mortality, and 2-year mortality after treatment with chemotherapy and best supportive care.

	Group	HR/RR and 95%CI	P value	Heterogeneity (%)	P value for heterogeneity
**Overall survival**	**Number of patients**				
	>200	0.80 (0.73–0.88)	<0.001	0	0.745
	<200	0.71 (0.60–0.84)	<0.001	36.8	0.124
	**Mean age**				
	>64	0.75 (0.64–0.87)	<0.001	29.7	0.223
	<64	0.79 (0.67–0.93)	0.005	44.9	0.106
	**Gender (male, %)**				
	>80	0.76 (0.64–0.92)	0.004	0	0.747
	<80	0.76 (0.66–0.86)	<0.001	45.1	0.068
	**Intervention**				
	Platinum-based chemotherapy	0.75 (0.65–0.86)	<0.001	37.2	0.121
	Gemcitabine/vinorelbine	0.79 (0.57–1.09)	0.154	54.5	0.138
	Taxel	0.68 (0.48–0.97)	0.034	-	-
	Pemetrexed	0.79 (0.65–0.96)	0.015	-	-
	**Jadad score**				
	>4	0.77 (0.68–0.86)	<0.001	0	0.640
	<4	0.76 (0.65–0.87)	<0.001	39.5	0.094
**12-monthmortality**	**Number of patients**				
	>200	0.90 (0.86–0.95)	<0.001	0	0.872
	<200	0.85 (0.78–0.93)	<0.001	17.4	0.301
	**Mean age**				
	>64	0.89 (0.84–0.95)	<0.001	5.4	0.376
	<64	0.88 (0.82–0.95)	0.001	9.2	0.354
	**Gender (male, %)**				
	>80	0.87 (0.79–0.96)	0.006	0	0.439
	<80	0.89 (0.85–0.93)	<0.001	0.5	0.425
	**Intervention**				
	Platinum-based chemotherapy	0.88 (0.83–0.93)	<0.001	0.6	0.412
	Gemcitabine/vinorelbine	0.88 (0.74–1.06)	0.172	63.5	0.098
	Taxel	0.90 (0.82–0.99)	0.028	0	0.713
	Pemetrexed	-	-	-	-
	**Jadad score**				
	>4	0.87 (0.81–0.93)	<0.001	0	0.570
	<4	0.90 (0.85–0.95)	<0.001	2.7	0.409
**2-year mortality**	**Number of patients**				
	>200	0.96 (0.91–1.01)	0.125	74.8	0.008
	<200	0.93 (0.84–1.02)	0.115	54.9	0.109
	**Mean age**				
	>64	0.96 (0.89–1.03)	0.282	65.1	0.057
	<64	0.93 (0.86–1.01)	0.098	80.3	0.006
	**Gender (male, %)**				
	>80	0.89 (0.83–0.95)	<0.001	-	-
	<80	0.97 (0.93–1.01)	0.095	47.9	0.087
	**Intervention**				
	Platinum-based chemotherapy	0.96 (0.92–1.00)	0.046	48.1	0.103
	Gemcitabine/vinorelbine	1.01 (0.96–1.08)	0.644	-	-
	Taxel	0.89 (0.83–0.95)	<0.001	-	-
	Pemetrexed	-	-	-	-
	**Jadad score**				
	>4	0.95 (0.91–0.99)	0.012	-	-
	<4	0.95 (0.90–1.00)	0.068	68.2	0.008

We used Egger test [16] to check for potential publication bias, which showed no evidence of publication bias for the outcomes of OS (p value, 0.226), 12-month mortality (p value, 0.093) and 2-year mortality (p value, 0.217).

## Discussion

In 2007, as a result of a meta-analysis of the third-generation chemotherapeutic agents in the treatment of NSCLC [35], chemotherapy promised to become a significant advancement in the treatment of NSCLC. Another meta-analysis conducted by the NSCLC Collaborative Group concluded that treatment with chemotherapy plus BSC improved OS compared to best supportive care alone by 23% [36]. In this updated comprehensive quantitative review, we included more than 4135 patients with NSCLC with a broad range of baseline characteristics. We have used strict inclusion criteria to limit comparisons to the effect of chemotherapy in the treatment of NSCLC. Although this is not an individual data meta-analysis, and therefore, the comparison of survival is based on the number of deaths computed from a pooled risk difference, our current study suggests that chemotherapy plus BSC can prolong OS, and reduces the risks of 6-month mortality, 12-month mortality, and 2-year mortality. Since nearly all the trials in our study included patients with stage III/IV disease or advanced NSCLC, the conclusions should be applicable only to patients with advanced or metastatic NSCLC.

We found that the treatment with chemotherapy plus BSC yielded a statistically significant benefit in OS as compared with BSC alone. However, our subgroup analysis suggested that gemcitabine/vinorelbine therapy did not have an effect on OS as compared to BSC (HR, 0.79; 95%CI, 0.57–1.09). These conclusions may be unreliable because of a small number of trials (2 trials). Additionally, the trials by ELCVIS Group [18] suggested that treatment with vinorelbine plus BSC was associated with a statistically significant improvement in OS compared with BSC in patients with NSCLC (HR, 0.65; 95%CI, 0.45–0.93). However, the trial by H Anderson et al. [17] indicated that treatment with gemcitabine plus BSC did not have an effect on OS as compared with BSC alone (HR, 0.91; 95%CI, 0.70–1.17). The reasons for this might be as follows: (1) The efficacy of gemcitabine was greater in men than in women (2) Vinorelbine might have direct beneficial effects on OS in patients with NSCLC, these effects may be reduced or balanced by gemcitabine. (3) A few trials were included in such subsets and only 2 trials reported gemcitabine/vinorelbine therapy.

Previous meta-analysis [36] failed to demonstrate the efficacy of chemotherapy on the risk of short-term or long-term mortality. However, in our study chemotherapy plus BSC reduced 6-month, 12-month, and 2-year mortality risks as compared to BSC alone. Similarly, we noted that gemcitabine/vinorelbine therapy did not have an effect on 12-month mortality; the reasons for this have already been discussed above. However, it should be noted that the 2-year mortality were not reduced when percentage male less than 80% and the treatment was gemcitabine/vinorelbine therapy. The reason for this could be that the short follow-up in some trials made it difficult to comment on the effect of treatment on the 2-year mortality. Another potential explanation could be that the effects of chemotherapy on the risk of long-term mortality in men were superior to the effects observed in women.

As expected, the toxicity was significantly more severe in patients who received chemotherapy plus BSC. Symptomatic improvements due to the tumor shrinkage should be balanced with increased toxic effects of chemotherapy, and concerns remain regarding the impact of the increased toxicity of chemotherapy on patients' qualities of life. The increased risks of neutropenia, leukopenia, anemia, infection, nausea/vomiting, alopecia, and ankle swelling were detected in patients treated with chemotherapy plus BSC in our study. In addition, it should be noted that chemotherapy plus BSC significantly increased the risk of hematological toxicities.

The purpose of undertaking this review was to determine whether chemotherapy plus BSC would improve survival, and to present all available evidence in a systematic, quantitative and an unbiased fashion. The findings of this study demonstrated that treatment with chemotherapy plus BSC was associated with a statistically significant improvement in OS compared with BSC alone. Additionally, it had a clear effect on the 6-month, 12-month and 2-year mortality. Several technical limitations of this meta-analysis should be acknowledged. First, inherent assumptions were made for all meta-analyses, because the analyses used pooled data, either published or provided by the individual study; individual patient data or original data were unavailable, which did not allow us to perform more detailed analyses and to obtain more comprehensive results. Second, treatments given in those trials included second generation, third generation, and the fourth generation chemotherapy regiments, which prevented us from exploring the association between the type of chemotherapy and survival outcomes. Third, heterogeneity among the trials is another limitation of our study. We applied a random-effect model that took possible heterogeneity into consideration and performed subgroup analyses based on several important factors to further explore the source of heterogeneity. Fourth, data on progression-free survival were rarely available in these trials; therefore, no conclusions could be drawn.

In future trials, it will be important to focus on the effects of chemotherapy on the risk of progression-free survival and to explore the efficacy of chemotherapy plus BSC on the disease recurrence as compared to BSC alone. We suggest that the ongoing trials should be improved in the following ways. First, the adverse toxicity in clinical trials should be recorded and reported normatively, so that the side effects of any treatments can be evaluated in future trials. Second, the progression-free survival should contain more details. Third, the role of treatment duration, the type of chemotherapy, and the dosage should be investigated in more detail to establish the optimal dose and treatment approaches.

## Supporting Information

Table S1 **PRISMA Checklist.**
(DOC)

Figure S1 **PRISMA Flowchart.**
(DOC)
