# Supplementary figures and images for: Chemotherapy Plus Best Supportive Care versus Best Supportive Care in Patients with Non-Small Cell Lung Cancer: A Meta-Analysis of Randomized Controlled Trials
Source: PLoS One. 2013 Mar 13;8(3):e58466. doi: 10.1371/journal.pone.0058466 (PMC3603584; doi:10.1371/journal.pone.0058466)

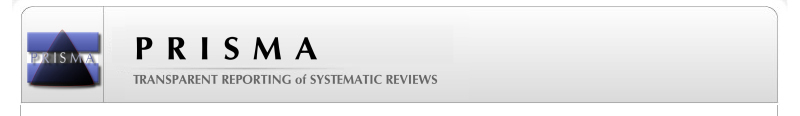
**PRISMA 2009 Flow Diagram**

**Screening**

**Included**

**Eligibility**

**Identification**

Supplement: Figure S1 — PRISMA Flowchart. (DOC) [file pone.0058466.s002.doc]
